# Plasma phospholipid transfer protein activity is inversely associated with betaine in diabetic and non-diabetic subjects

**DOI:** 10.1186/s12944-016-0313-5

**Published:** 2016-08-31

**Authors:** R. P. F. Dullaart, Erwin Garcia, Elias Jeyarajah, Eke G. Gruppen, Margery A. Connelly

**Affiliations:** 1 Department of Endocrinology, University Medical Center Groningen, University of Groningen, P.O. Box 30.001, Groningen, 9700 RB The Netherlands; 2 LipoScience, Laboratory Corporation of America® Holdings, Raleigh, NC USA; 3 Departments of Endocrinology and Nephrology, University of Groningen and University Medical Center Groningen, Groningen, The Netherlands

**Keywords:** Betaine, Lecithin:cholesterol acyltransferase, Lipoproteins, Phospholipid transfer protein, Type 2 diabetes mellitus

## Abstract

**Background:**

The choline metabolite, betaine, plays a role in lipid metabolism, and may predict the development of cardiovascular disease and type 2 diabetes mellitus (T2DM). Phospholipid transfer protein (PLTP) and lecithin:cholesterol acyltransferase (LCAT) require phosphatidylcholine as substrate, raising the possibility that there is an intricate relationship of these protein factors with choline metabolism. Here we determined the relationships of PLTP and LCAT activity with betaine in subjects with and without T2DM.

**Methods:**

Plasma betaine (nuclear magnetic resonance spectroscopy), PLTP activity (liposome-vesicle HDL system), LCAT activity (exogenous substrate assay) and (apo)lipoproteins were measured in 65 type 2 diabetic (T2DM) and in 55 non-diabetic subjects.

**Results:**

PLTP and LCAT activity were elevated in T2DM (*p* < 0.05), whereas the difference in betaine was not significant. In age-, sex- and diabetes status-controlled correlation analysis, betaine was inversely correlated with triglycerides and positively with HDL cholesterol (*p* < 0.05 to 0.01). PLTP and LCAT activity were positively correlated with triglycerides and inversely with HDL cholesterol (*p* < 0.05 to 0.001). PLTP (*r* = −0.245, *p* = 0.006) and LCAT activity (*r* = −0.195, *p* = 0.035) were correlated inversely with betaine. The inverse association of PLTP activity with betaine remained significant after additional adjustment for body mass index and lipoprotein variables (β = −0.179, *p* = 0.034), whereas its association with LCAT activity lost significance (β = −0.056, *p* = 0.44).

**Conclusions:**

Betaine may influence lipoprotein metabolism via an effect on PLTP activity.

## Background

Choline is regarded as a semi-essential nutrient which originates from both dietary sources and from *de novo* production in the human body [1]. Choline is to an important extent oxidized to betaine [1]. Among other effects, betaine is intricately involved in lipid metabolism as evidenced by its ability to attenuate hepatic triglyceride accumulation [1]. In humans, plasma betaine may relate inversely to triglycerides and non-high density lipoprotein (non-HDL) cholesterol, and positively to HDL cholesterol [1–3]. In addition, choline is a component of phosphatidylcholine, a key phospholipid constituent of lipoproteins and cell membranes [1].

Evidence has accumulated recently that abnormalities in the choline pathway may be relevant for the development of cardiometabolic disorders [4–7]. Thus, low betaine levels may predict cardiovascular disease (CVD) in non-diabetic subjects, and modify the extent to which trimethylamine-N-oxide (TMAO), a metabolite that results from intestinal choline metabolism, confers an increased CVD risk [4, 5]. Low betaine levels may also be associated with increased incidence of type 2 diabetes mellitus (T2DM) [6, 7].

Among other factors, human lipoprotein metabolism is governed by phospholipid transfer protein (PLTP), which transfers phospholipids, in particular phosphatidylcholine, between lipoproteins and is crucial for HDL remodelling [8, 9], as well as by lecithin:cholesterol acyltransferase (LCAT) which generates cholesteryl esters by using phosphatidylcholine as a substrate [10, 11]. Both PLTP and LCAT thus play a pivotal role in HDL metabolism. Interestingly, in vivo administration of artificial phosphatidylcholine-apolipoprotein A-I containing particles enhances the activities of both PLTP and LCAT [12], suggesting that the activity of these proteins is in part dependent on phosphatidylcholine. Collectively, these findings [8–10, 12] raise the possibility that choline metabolites may relate to PLTP and LCAT activity. Furthermore, both PLTP and LCAT activity have been found to be elevated in T2DM [9, 11], making the diabetic state a relevant condition to assess their relationships with betaine.

Here we determined the extent to which plasma PLTP and LCAT activity are associated with betaine levels in subjects with and without T2DM.

## Methods

### Subjects

The study protocol was approved by the medical ethics committee of the University Medical Center Groningen. T2DM subjects, aged >18 years, participated after written informed consent had been obtained. T2DM was diagnosed previously by primary care physicians based on a fasting plasma glucose ≥7.0 mmol/L and/or non-fasting plasma glucose ≥11.1 mmol/L). The use of metformin, sulfonylurea and antihypertensive medication was allowed, but insulin use was an exclusion criterion. Current smokers and subjects who used lipid lowering drugs were also excluded, as were subjects with a history of CVD, chronic kidney disease (estimated glomerular filtration rate < 60 mL/min/1.73 m^2^ and/or proteinuria), liver function abnormalities or thyroid dysfunction. Blood pressure was measured after 15 min. rest at the left arm in sitting position using a sphygmomanometer. Body mass index (BMI in kg/m^2^) was calculated as weight divided by height squared. Elevated blood pressure, low HDL cholesterol and elevated triglycerides were categorized according to NCEP-ATPIII metabolic syndrome criteria.

Sixty-five diabetic subjects and 55 non-diabetic subjects participated (Table 1). Mean diabetes duration was 5.4 ± 2.1 years. Fourteen T2DM patients used metformin and 13 used sulfonylurea alone. Both drugs were used by 19 patients. No other glucose lowering drugs were taken. Anti-hypertensive medication, particularly angiotensin-converting enzyme inhibitors, angiotensin II receptor antagonists and diuretics, alone or in combination) were used by 27 T2DM subjects. None of the non-diabetic subjects used anti-hypertensive drugs (*p* < 0.001). Three non-diabetic women (2 post- and 1 premenopausal women) used estrogens. Other medications were not used.

**Table 1:** Clinical characteristics, plasma (apo)lipoproteins, betaine, phospholipid transfer protein (PLTP) activity and lecithin:cholesterol acyltransferase (LCAT) activity in 65 Type 2 diabetic (T2DM) patients and in 55 non-diabetic subjects, and partial correlation coefficients with betaine, PLTP activity and LCAT activity in whole study group

			Partial correlation coefficient of variable with		
	T2DM subjects (*n* = 65)	Non-diabetic subjects (*n* = 55)	Betaine	PLTP activity	LCAT activity
Age (years)	59 ± 9**	54 ± 10			
Sex (men/women)	40/25*	23/32			
Systolic blood pressure (mm Hg)	146 ± 20***	130 ± 20	−0.04	0.033	0.033
Diastolic blood pressure (mm Hg)	87 ± 9*	82 ± 11	−0.172	0.061	0.237*
BMI (kg/m^2^)	28.9 ± 4.9***	25.8 ± 4.1	−0.036	0.229*	0.242*
Glucose (mmol/L)	8.95 ± 2.33***	5.65 ± 0.62	−0.225*	0.171	0.390***
HbA1c (mmol/mol)	50 ± 5***	34 ± 2	−0.245**	0.199*	0.306***
Total cholesterol (mmol/L)	5.34 ± 0.92*	5.76 ± 0.97	−0.144	0.225*	0.544***
Non-HDL cholesterol (mmol/L)	4.04 ± 1.09	4.23 ± 1.05	−0.209*	0.281**	0.590***
LDL cholesterol (mmol/L)	3.21 ± 0.84	3.50 ± 0.86	−0.139	0.167	0.457***
HDL cholesterol (mmol/L)	1.30 ± 0.39**	1.53 ± 0.40	0.218*	−0.213*	−0.265**
Triglycerides (mmol/L)	1.73 (1.20–2.17)	1.37 (0.88–1.92)	−0.239**	0.312***	0.614***
ApoB (g/L)	0.92 ± 0.23	0.95 ± 0.24	−0.138	0.252**	0.493***
ApoA-I (g/L)	1.36 ± 0.24	1.43 ± 0.21	0.057	−0.139	0.040
Betaine (μmol/L)	24.5 ± 10.3	26.7 ± 9.0			
PLTP activity (AU)	104.3 ± 11.6***	94.5 ± 10.5	−0.254**		
LCAT activity (AU)	114.5 ± 17.7*	107.6 ± 13.3	−0.195*	0.135	

Data are means ± SD or medians (interquartile range)

*BMI* body mass index, *HbA1c* glycated hemoglobin, *apo* apolipoprotein, *HDL* high density lipoproteins, *LDL* low density lipoproteins. LDL cholesterol was calculated in 62 T2DM subjects and in 53 non-diabetic subjects. Partial correlation coefficients controlling for age, sex and diabetes status are shown

*p < 0.05; **p ≤ 0.01; ***p ≤ 0.001

### Laboratory methods

Serum and EDTA-anticoagulated plasma samples were stored at −80 °C until analysis. Plasma glucose was measured shortly after blood collection.

Total cholesterol and triglycerides were measured by routine enzymatic methods (Roche/Hitachi cat. nos 11875540 and 1187602, respectively; Roche Diagnostics GmBH, Mannheim, Germany). HDL cholesterol was assayed by a homogeneous enzymatic colorimetric test (Roche/Hitachi, cat.no 04713214). Non-HDL cholesterol was calculated as the difference between total cholesterol and HDL cholesterol. Low density lipoprotein (LDL) cholesterol was calculated by the Friedewald formula if triglycerides were < 4.5 mmol/L. Apolipoprotein A-I (apoA-I) and apolipoprotein B (apoB) were measured by immunoturbidimetry (Roche/Cobas Integra Tinaquant cat no. 03032566, Roche Diagnostics). Glucose was analyzed with an APEC glucose analyzer (APEC Inc., Danvers, MA). HbA1c was measured by high-performance liquid chromatography (Bio-Rad, Veenendaal, the Netherlands; normal range: 27–43 mmol/mol).

PLTP activity (expressed in arbitrary units [AU] relative to pool plasma) was measured with a liposome vesicles–HDL system [9]. The intra-assay coefficient of variation of these assays is less than 5 %. Plasma LCAT activity (expressed in AU relative to pool plasma) was determined using excess exogenous substrate containing [^3^H]-cholesterol [11]. Samples were incubated with labelled substrate for 6 h at 37 °C. Corrections were made for the amount of free cholesterol in the plasma samples. The reaction was stopped by addition of cold ethanol to the incubation medium. Free and esterified cholesterol were separated using disposable silica columns. [^3^H]-cholesteryl esters were eluted with hexane. Plasma LCAT activity is strongly correlated with LCAT concentration. The intra- and interassay coefficients of variation for PLTP and LCAT activity are < 5 %.

Betaine was measured by ^1^H-nuclear magnetic resonance (NMR) spectroscopy using a Vantera® NMR Clinical Analyzer (LabCorp, Raleigh, NC). Plasma specimens were diluted with citrate/phosphate buffer (3:1 v/v) to lower the pH to 5.3 in order to separate the betaine and TMAO signals which overlap at physiological pH. [^1^H]-NMR spectra were acquired using the Car-Purcell-Meiboom-Gill (CPMG) acquisition technique on a 400 MHz spectrometer (48 scans). The betaine peak at 3.22 ppm was quantified using a proprietary bound-constrained non-linear least squares algorithm that models the lineshape as log-normal and lorentzian peak shapes. The derived betaine signal amplitudes were converted to μmol/L units using a factor that was empirically determined using dialyzed serum samples spiked with known amounts of betaine. The betaine quantitation software was validated using pooled sera with known amounts of betaine prepared by serial dilution. The measured betaine values agreed well with expected concentrations (slope = 0.998, intercept = 0.890, r^2^ = 0.996). The intra- and interassay coefficients of variation for betaine are 6.2 and 7.2 %, respectively.

### Statistical analysis

SPSS (version 22.0, SPSS Inc. Chicago, IL, USA) was used for data analysis. Results are expressed as mean ± SD or as median (interquartile range). Because of skewed distribution, logarithmically transformed values of triglycerides were used. Between group differences in variables were determined by unpaired t-tests or by Chi-square tests. Univariate correlations were determined by Pearson correlation coefficients. Partial correlation coefficients were calculated controlling for age, sex and diabetes status. Multivariable linear regression analyses were carried out to disclose the independent relationships of PLTP and LCAT activity with betaine. Two-sided *p*-values <0.05 were considered significant.

## Results

T2DM subjects were older and more likely to be men (Table 1). Systolic and diastolic blood pressure, BMI, glucose and HbA1c were higher in T2DM subjects. Total cholesterol was lower in T2DM which was in part attributable to lower HDL cholesterol. Triglycerides tended to be elevated in T2DM (*p* = 0.081). High blood pressure was more prevalent in T2DM vs. non-diabetic subjects (*p* < 0.001), but the difference in low HDL cholesterol (*p* = 0.074) and elevated triglycerides (*p* = 0.13) did not reach significance. Non-HDL cholesterol, LDL cholesterol, apoB and apoA-I levels were not significantly different between the groups. Betaine tended to be lower in T2DM but the difference was not significant (*p* = 0.20). PLTP and LCAT activity were each increased in T2DM subjects (Table 1). Betaine, PLTP and LCAT activity were not different between men and women (*p* > 0.10 for each; data not shown). In the whole group, betaine (*r* = −0.144, *p* = 0.12) and LCAT activity (*r* = 0.072, *p* = 0.43) were unrelated to age, but PLTP activity tended to be higher with advancing age (*r* = 0.164, *p* = 0.073).

In the whole group, betaine was correlated inversely with PLTP activity (*r* = −0.307, *p* = 0.001) and with LCAT activity (*r* = −0.223, *p* = 0.014) in univariate analysis. In T2DM subjects separately, betaine also (tended to be) inversely related to PLTP activity (*r* = −0.447, *p* < 0.001) and LCAT activity (*r* = −0.238, *p* = 0.056). These relationships were non-significant in non-diabetic subjects (*r* = −0.046, *p* = 0.75 and *r* = −0.145, *p* = 0.29, respectively). After controlling for age, sex and diabetes status, betaine was correlated inversely with glycemia, non-HDL cholesterol and triglycerides, and positively with HDL cholesterol (Table 1). PLTP and LCAT activity were correlated positively with BMI, glycemia, total cholesterol and non-HDL cholesterol, triglycerides and apoB, and inversely with HDL cholesterol. Betaine was correlated inversely with both PLTP and LCAT activity (Table 1).

Multivariable linear regression analyses were carried out to discern the independent associations PLTP activity and LCAT activity with betaine (Table 2). In analyses in which we adjusted for age, sex, diabetes status, BMI and lipoprotein variables (representing variables to which betaine, PLTP and/or LCAT activity were correlated in age-, sex- and diabetes status-adjusted analysis as shown in Table 1), PLTP activity was independently and inversely associated with betaine (Table 2A, model 1). A similar inverse association was found when lipoprotein variables were replaced by apoB and apoA-I (Table 2A, model 2). These relationships remained essentially unaltered after additional adjustment for the use of glucose lowering drugs or antihypertensive medication (β = −0.204 to −0.16, *p* = 0.015 to *p* = 0.052; data not shown). Of note, the association of LCAT activity with betaine was no longer significant after adjustment for age, sex, diabetes status, BMI and lipoprotein variables or apolipoproteins (Table 2B, models 1 and 2). This association was also not significant after additional adjustment for glucose lowering drugs or antihypertensive medication use (β = −0.138 to −0.044, *p* = 0.093 to *p* = 0.54; data not shown).

**Table 2:** Multivariable linear regression analysis showing associations of plasma phospholipid transfer protein (PLTP) activity (A) and lecithin:cholesterol acyltransferase (LCAT) activity (B) as dependent variables with clinical variables, (apo)lipoproteins and betaine as statistical determinants

	Model 1		Model 2	
	β	*p*-value	β	*p*-value
A. PLTP activity				
Age	0.076	0.37	0.078	0.36
Sex (men/women)	−0.139	0.14	−0.163	0.07
Diabetes (yes/no)	0.340	<0.001	0.339	<0.001
BMI	0.176	0.06	0.181	0.043
Non-HDL cholesterol	0.126	0.22		
HDL cholesterol	0.064	0.59		
Triglycerides	0.146	0.232		
Apolipoprotein B			0.166	0.05
Apolipoprotein A-I			−0.014	0.88
Betaine	−0.179	0.034	−0.204	0.014
B. LCAT activity				
Age	0.007	0.92	−0.003	0.97
Sex (men/women)	0.004	0.96	−0.006	0.95
Diabetes (yes/no)	0.186	0.018	0.188	0.034
BMI	0.154	0.053	0.217	0.013
Non-HDL cholesterol	0.344	<0.001		
HDL cholesterol	0.304	0.003		
Triglycerides	0.505	<0.001		
Apolipoprotein B			0.490	<0.001
Apolipoprotein A-I			0.198	0.033
Betaine	−0.056	0.44	−0.130	0.10

*β* standardized regression coefficients, *BMI* body mass index, *HDL* high density lipoproteins. Triglycerides levels are logarithmically transformed

Model 1: includes age, sex, diabetes status, BMI, plasma non-HDL cholesterol, HDL cholesterol, triglycerides and betaine as independent variables

Model 2: includes age, sex, diabetes status, BMI, apolipoprotein B, apolipoprotein A-I and betaine as independent variables

## Discussion

In this study, we used a newly developed NMR spectrocopy-based assay to determine plasma betaine. Plasma betaine was correlated inversely with triglycerides and positively with HDL cholesterol. In contrast, PLTP and LCAT activity were correlated positively with triglycerides and inversely with HDL cholesterol. Both PLTP and LCAT activity were correlated inversely with betaine in univariate and in age-, sex- and diabetes status-adjusted analysis, but only the relationship with PLTP activity remained statistically significant in analysis in which we additionally controlled for BMI and (apo)lipoprotein variables. Our findings raise the possibility that betaine, an oxidation product of choline metabolism, could influence lipoprotein metabolism via an effect on PLTP activity.

Given the relevance of PLTP and LCAT for altered HDL remodelling and triglyceride metabolism in diabetes [9–11, 13], we decided to include both diabetic and non-diabetic subjects in the present study. Plasma PLTP and LCAT activity were expectedly elevated in T2DM, and both protein factors were correlated positively with triglycerides [9, 11, 13, 14]. Betaine was not significantly different between diabetic and non-diabetic subjects, despite being inversely correlated with glucose and HbA1c. In comparison, betaine has been variably reported to be lower [6] or unchanged in T2DM [15].

In keeping with earlier reports, betaine was related inversely to triglycerides and non-HDL cholesterol, and positively with HDL cholesterol [1–3]. Of note, both betaine and PLTP affect hepatic triglyceride metabolism although by different mechanisms [16, 17]. Moreover, not only plasma betaine but also PLTP activity has been suggested to predict incident T2DM [6, 7, 14]. Thus, in view of the presently documented inverse relationship between PLTP and betaine, it seems warranted to take both factors into consideration when evaluating the extent to which they may confer cardiometabolic risk [4, 5, 18].

Several methodological considerations and limitations of our study need to be appreciated. First, we conducted a cross-sectional study in a rather restricted number of participants. Thus, cause-effect relationships cannot be established with certainty. Second, we excluded subjects who used lipid lowering treatment. As a result, it is likely that diabetic subjects with mild lipoprotein abnormalities were preferentially included, limiting extrapolation of the current findings to subjects with more severe dyslipidemia. Third, it is obvious that the explorative design of our study necessitates to delineate more precisely the metabolic interplay between the generation of betaine from choline on the one hand and the conversion of choline to phosphatidylcholine on the other.

## Conclusions

Our present results are consistent with the concept that the choline metabolite, betaine, may influence lipoprotein metabolism via an effect on PLTP activity.

## Abbreviations

ApoA-I: Apolipoprotein A-I
ApoB: Apolipoprotein B
BMI: Body mass index
CVD: Cardiovascular disease
HDL: High density lipoprotein
LCAT: Lecithin:cholesterol acyltransferase
LDL: Low density lipoprotein
PLTP: Phospholipid transfer protein
T2DM: Type 2 diabetes mellitus
TMAO: Trimethylamine-N-oxide
